# The Bork-Baykal Phenomenon in Congenital Melanocytic Nevus

**DOI:** 10.5826/dpc.1103a58

**Published:** 2021-07-08

**Authors:** Tugba Kevser Uzuncakmak, Defne Özkoca, Server Serdaroğlu

**Affiliations:** Istanbul University-Cerrahpasa, Cerrahpasa Medical Faculty Department of Dermatology

**Keywords:** Baykal Phenomenon, congenital nevus, melanocytic nevus

## Case Presentation

**Figure 1:**
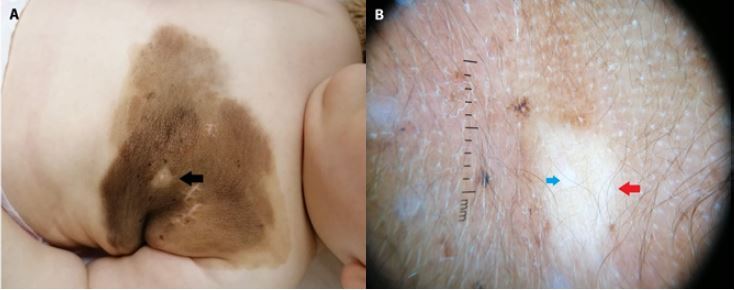
(A) 15×15 cm sized, light-dark brown, multicomponent CMN with two excision scars over the left breast without the involvement of the nipple and areola. (B) Whitish homogeneous area surrounded by brown homogeneous area and dark brown, symmetrical reticular lines.

A 6-month-old girl was admitted to our outpatient clinic for dermoscopic examination of a large congenital melanocytic nevus on chest (CMN). Dermatological examination revealed 15×15 cm sized, light-dark brown, multicomponent CMN with 2 excision scars over the left breast. Nipple and areola were not involved (Figure 1A). Dermoscopic examination revealed a whitish homogenous area surrounded by a brown homogenous area and dark brown, symmetrical reticular lines (Figure 1B).

## Teaching Point

Sparing of the nipple-areola complex (the Bork–Baykal phenomenon) was first reported by Baykal et al in 8 cases of large congenital melanocytic nevus (CMN) sparing the areola [1]. 2 years later, Happle referred to this entity as “the Bork-Baykal phenomenon: a new and rarely seen entity referring to the nipple-sparing nevus of the breast”. Medium and large CMNs are a subtype of hamartomas that present at birth. Their peculiar appearance was linked to the different histopathological features and embryologic developmental periods of the affected tissues and the nipple-areola complex [2].
